# Prevalence and Mortality of Hypochloremia Among Patients Suffering From Coronary Artery Disease and Congestive Heart Failure: An Analysis of Patients in CIN-I and MIMIC-III Databases

**DOI:** 10.3389/fmed.2021.769646

**Published:** 2021-12-21

**Authors:** Haozhang Huang, Jin Liu, Yan Liang, Kunming Bao, Linfang Qiao, Jiulin Liu, Qiang Li, Bo Wang, Shiqun Chen, Wenguang Lai, Cong Chen, Lingyu Zhang, Xiaoyu Huang, Dehua Huang, Jiyan Chen, Ning Tan, Yong Liu

**Affiliations:** ^1^Department of Cardiology, Guangdong Provincial Key Laboratory of Coronary Heart Disease Prevention, Guangdong Cardiovascular Institute, Guangdong Provincial People's Hospital, Guangdong Academy of Medical Sciences, Guangzhou, China; ^2^The Second School of Clinical Medicine, Southern Medical University, Guangzhou, China; ^3^Department of Cardiology, Maoming People's Hospital, Maoming, China; ^4^Department of Cardiology, Longyan First Affiliated Hospital of Fujian Medical University, Longyan, China; ^5^Guangdong Provincial People's Hospital, School of Biology and Biological Engineering, South China University of Technology, Guangzhou, China; ^6^Department of Cardiology, People's Hospital of Yangjiang, Yangjiang, China; ^7^Guangdong Provincial People's Hospital, School of Medicine, South China University of Technology, Guangzhou, China

**Keywords:** coronary artery disease, congestive heart failure, prevalence, mortality, hypochloremia

## Abstract

**Background:** Hypochloremia is an independent predictor for mortality in patients with coronary artery disease (CAD) but whether the same correlation exists in CAD patients with congestive heart failure (CHF) is unclear.

**Methods:** This is an analysis of data stored in the databases of the CIN-I [a registry of Cardiorenal Improvement (NCT04407936) in China from January 2007 to December 2018] and Medical Information Mart for Intensive Care (MIMIC)-III. CAD patients with CHF were included. The outcome measures were 90-day all-cause mortality (ACM) and long-term ACM.

**Results:** Data from 8,243 CAD patients with CHF were analyzed. We found that 10.2% of the study population had hypochloremia (Cl^−^ <98 mmol/L) in CIN-I (*n* = 4,762) and 20.1% had hypochloremia in MIMIC-III (*n* = 3,481). Patients suffering from hypochloremia were, in general, older and had a higher prevalence of comorbidities. After adjustment for confounders, hypochloremia remained a significant predictor of short-term mortality risk [90-day ACM: adjusted hazard ratio (a*HR*), 1.69; 95% CI, 1.27–2.25; *P* < 0.001 in CIN-I, and 1.36 (1.17–1.59); *P* < 0.001 in MIMIC-III]. Hypochloremia was also associated with long-term mortality [a*HR*, 1.26; 95% *CI*, 1.06–1.50; *P* = 0.009 in CIN-I, and 1.48 (1.32–1.66); *P* < 0.001 in MIMIC-III]. Prespecified subgroup analyses revealed an association of hypochloremia with long-term ACM to be attenuated slightly in the women of the two databases (*P* interaction < 0.05).

**Conclusions:** Hypochloremia is independently associated with higher short-term and long-term ACM. Further studies are needed to determine if early preventive measurements and active intervention of hypochloremia can reduce the mortality risk of CAD patients with CHF.

## Introduction

Coronary artery disease (CAD) is a leading cause of morbidity and mortality worldwide. Often, CAD is complicated with heart failure (HF), hypertension, and chronic kidney disease (CKD) (1, 2), and the mortality of patients with CAD is increased markedly if they also have HF (3). Congestive heart failure (CHF) describes an inadequacy of the pumping function of the heart (4), which is tightly linked to CAD (5).

Li and colleagues indicated that hypochloremia is associated with an increased risk of all-cause mortality (ACM) among patients in the Cardiac Care Unit (those with severe CAD) (6). Several studies have shown that hypochloremia is independently associated with mortality among patients with cardiovascular disease, especially those with acute HF or CHF even after taking the use of diuretics into account (7–10). Often, patients with CHF and CAD have electrolyte disorders, such as hypochloremia, which may be related to diuretic use, insufficient intake of fluids, or a complex association between diseases.

However, it is not clear if the same connection exists among CAD patients complicated with CHF after full consideration of diuretic use. The loss of gastrointestinal digestive juices, derangement of secretion of adrenal hormones, the inflammatory response, and the critical role of chloride ions (Cl^−^) in several regulatory pathways central to HF have been postulated (11–14).

In this context, we aimed to assess the prevalence and mortality (short- and long-term) of hypochloremia among CAD patients with CHF.

## Methods

### Data Collection

From January 2007 to December 2018, we accessed data from the Electronic Clinical Management System of Guangdong Provincial People's Hospital (Guangdong, China). The Cardiorenal Improvement (CIN)-I registry was registered at ClinicalTrials.gov (NCT04407936).

This was a single-center, observational, retrospective cohort study that included patients undergoing coronary angiography (15). Percutaneous coronary intervention (PCI) or coronary angiography was undertaken in accordance with the standard guidelines (16, 17). The information of patients at baseline was demographics, laboratory test results, and other clinical variables. Blood samples were collected in the early morning after an overnight fast.

**Figure 1:**
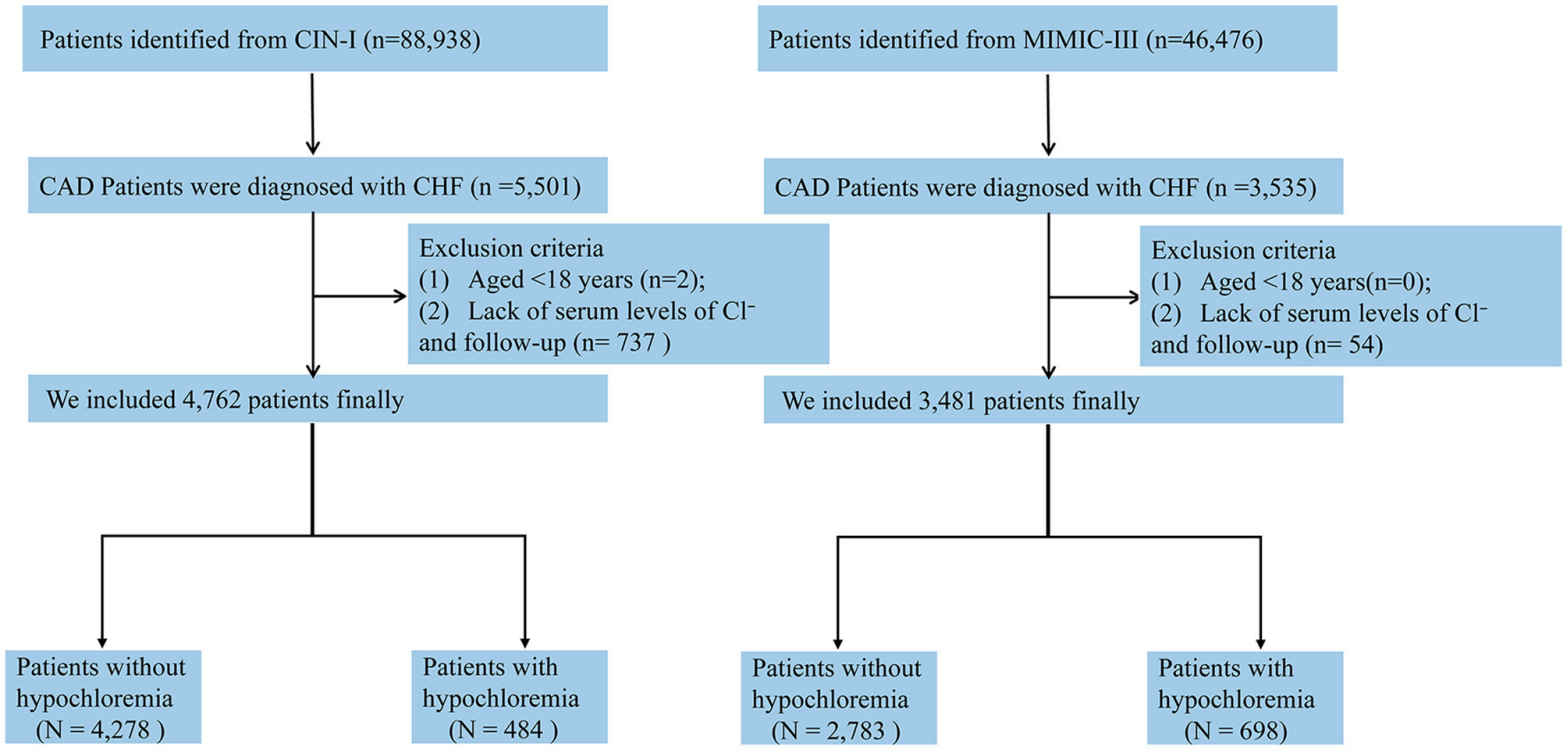
The flow of participants through the trial.

In addition, we utilized the Medical Information Mart for Intensive Care (MIMIC)-III database to undertake validation. The MIMIC database was developed and is maintained by the Laboratory for Computational Physiology at the Massachusetts Institute of Technology (Cambridge, MA, USA). MIMIC-III contains data from the patients in the intensive care unit (ICU) and includes physiologic information from bedside monitors in the adult ICUs of Beth Israel Deaconess Medical Center, a tertiary-care university hospital in Boston (MA, USA). The database includes information from 2002 to 2011 (Figure 1) (18).

### Study Population

**Figure 2:**
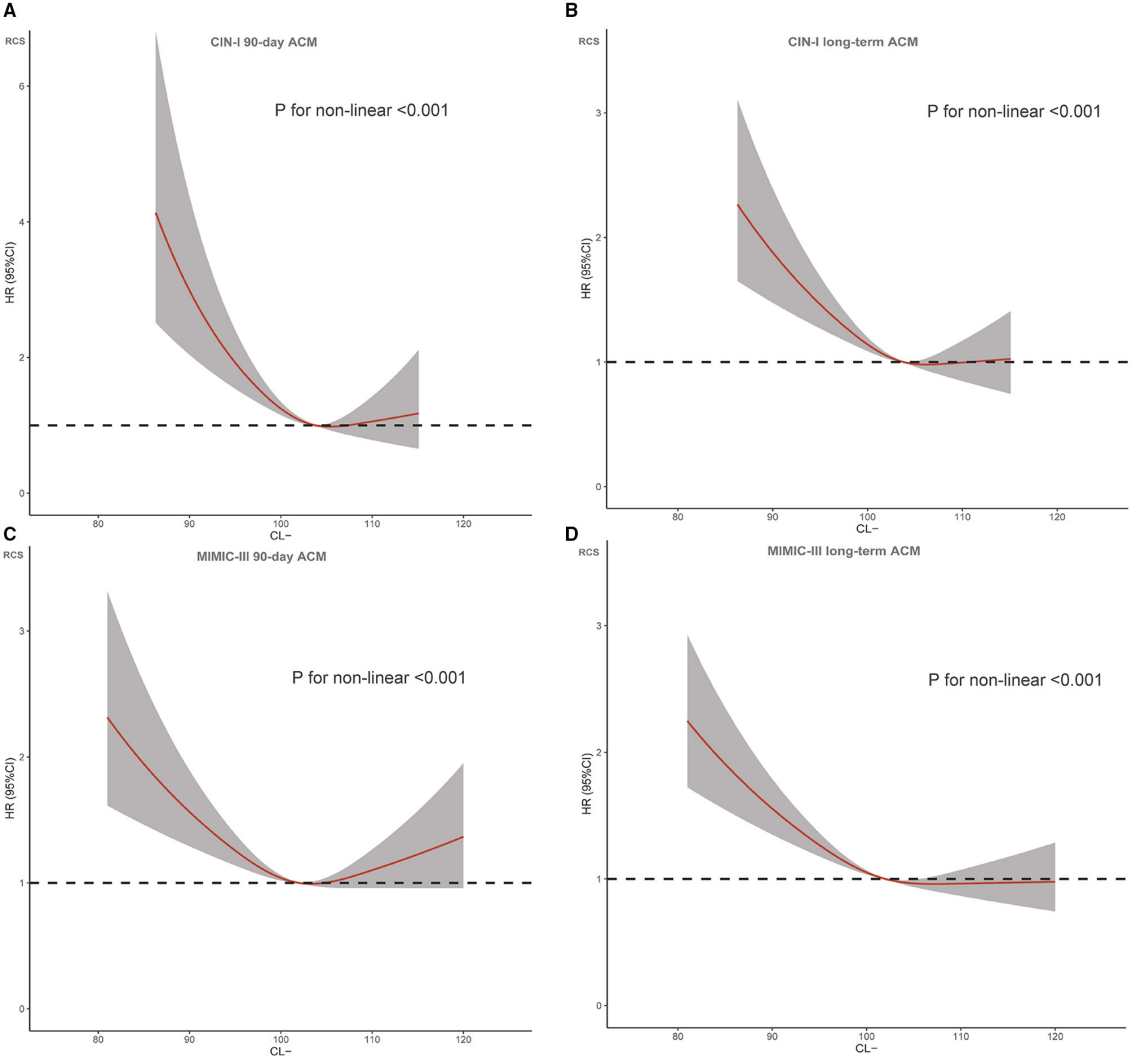
Restricted spline curve for the chloridion hazard ratio (*HR*). **(A)** CIN-I 90-day ACM; **(B)** CIN-I long-term ACM **(C)** MIMIC-III 90-day ACM; **(D)** MIMIC-III long-term ACM.

All CAD patients with CHF in the CIN-I and MIMIC-III databases were eligible for inclusion in our investigation. Two exclusion criteria were used: (i) age <18 years; and (ii) data for serum levels of Cl^−^ and follow-up were missing. Only the data of the first ICU admission of the first hospitalization in MIMIC-III were used. Accordingly, 4,762 CAD patients with CHF in CIN-I and 3,481 patients in MIMIC-III were enrolled (Figure 2).

### Endpoints and Definitions

The primary endpoints were 90-day ACM and long-term ACM after hospital admission. Follow-up information was accessed from Guangdong Provincial Public Security (which is paired with the Electronic Clinical Management System of Guangdong Provincial People's Hospital).

A Cl^−^ concentration in serum <98 mmol/L denoted hypochloremia. CAD was diagnosed according to the 10th Revision Codes of the International Classification of Diseases (ICD-10; I20.xx–I25.xx, I50.00001, and I91.40001) in the CIN-I database and ICD-9 codes in the MIMIC-III database. “CHF” was defined as New York Heart Association class > 2 or Killip class > 1 (19). “CKD” was defined as an estimated glomerular filtration rate ≤ 60 ml/min/1.73 m^2^. “Anemia” was defined as hematocrit <36% for women and <39% for men (19). Information on comorbidities, such as atrial fibrillation, chronic obstructive pulmonary disease (COPD), and stroke, was collected for the analyses based on the recorded ICD-9 codes in the MIMIC-III database and ICD-10 codes in the CIN-I database.

### Statistical Analyses

Descriptive statistics are given as the mean (SD), or numbers and percentages. Continuous variables were compared using the Student's *t*-test or Wilcoxon rank sum test, as appropriate. The chi-squared test was used to analyze categorical data. Patients were categorized into groups according to the cohort assessed (MIMIC-III or CIN-I).

Kaplan–Meier curves displayed the time-to-event data. Survival between groups was compared using log-rank tests. Restricted cubic splines were employed to inquire into the association between the serum level of Cl^−^ and 90-day ACM and long-term ACM. Cox regression models for 90-day ACM and long-term ACM were fitted (**Table 2**) to evaluate the association between hypochloremia and study endpoints. Model 1 was unadjusted. Model 2 adjusted for age (as a continuous variable) and sex. Model 3 included the variables which were associated with mortality according to clinical experience {demographic characteristics (age and sex) and medical history [hypotension, anemia, diabetes mellitus (DM), atrial fibrillation, CKD, COPD, stroke, and acute myocardial infarction (AMI)]}. Ultimately, we took the results of model 3 as the primary results.

### Subgroup and Sensitivity Analyses

To describe our results succinctly, we adopted COX regression models for 90-day ACM and long-term ACM. To avoid bias introduced by different models, the analysis of the primary outcome was replicated in a logistic regression model for 90-day ACM (Supplementary Table 1). Finally, we undertook analyses among five pre-specified subgroups [age (>65 or ≤ 65 years), sex (male or female), AMI (yes or no), use of diuretics (yes or no), and hyponatremia (yes or no)] to evaluate the impact of hypochloremia on long-term ACM among CAD patients with CHF. *P* < 0.05 (two-sided) was considered significant. Statistical analyses were undertaken using R 4.0.3 (R Institute for Statistical Computing, Vienna, Austria).

## Results

### Characteristics of Patients

The characteristics of CIN-I and MIMIC-III databases at baseline are shown in Table 1. Among CAD patients with CHF in CIN-I, the mean age was 65.5 ± 10.9 years, 24.6% were women, 2,192 (46.5%) had AMI, 2,063 (43.7%) CKD, and 3,458 (72.6%) underwent PCI. Among CAD patients with CHF in MIMIC-III, the mean age was 74.3 ± 11.6 years, 38.6% were women, 1,789 (51.4%) had AMI, 946 (27.2%) had CKD, and 654 (20.6%) underwent PCI.

**Table 1:** Baseline characteristics of the included patients in a registry of Cardiorenal Improvement (NCT04407936) (CIN-I) and Medical Information Mart for Intensive Care (MIMIC-III).

**Characteristic**	**CIN-I**	**MIMIC-III**
	**(*N* = 4,762)**	**(*N* = 3,481)**
**Demographic characteristics**		
Female, *n* (%)	1,173 (24.6)	1,344 (38.6)
Age, years, mean (SD)	65.5 (10.9)	74.3 (11.6)
**Medical history**		
Anemia, *n* (%)	2,192 (46.5)	2,486 (71.5)
AMI, *n* (%)	2,303 (48.4)	1,789 (51.4)
DM, *n* (%)	1,659 (34.8)	1,471 (42.3)
CKD, *n* (%)	2,063 (43.7)	946 (27.2)
Hypertension, *n* (%)	2,791 (58.6)	1,397 (40.1)
AF, *n* (%)	360 (7.6)	1,535 (44.1)
COPD, *n* (%)	79 (1.7)	199 (5.7)
Stroke, *n* (%)	394 (8.3)	374 (10.7)
**Procedure**, ***n*** **(%)**		
PCI	3,458 (72.6)	654 (20.6)
DES	3,293 (69.2)	320 (10.1)
BES	88 (1.8)	369 (11.6)
**Laboratory tests**		
NT-proBNP, pg/ml, mean (SD)	5,016.1 (9,491.5)	13,784.16 (16,664.6)
HGB, g/L, mean (SD)	127.2 (20.5)	115.3 (21.2)
eGFR, mL/min/1.73 m^2^ [mean (SD)]	65.2 (27.4)	55.3 (31.8)
K, mmol/L, mean (SD)	3.84 (0.54)	4.41 (0.82)
Na, mmol/L, mean (SD)	138.2 (3.6)	138.0 (4.7)
Ca, mmol/L, mean (SD)	2.20 (0.14)	2.16(0.21)
**Medications**		
ACEI/ARB, *n* (%)	2,116 (47.8)	1,949 (56.0)
Beta-blockers, *n* (%)	3,511 (79.3)	2,903 (83.4)
CCB, *n* (%)	785 (17.7)	836 (24.0)
Statin, *n* (%)	4,024 (90.9)	2,620 (75.3)
Aspirin, *n* (%)	3,861 (87.2)	2,827 (81.2)
Clopidogrel, *n* (%)	3,628 (82.0)	1,452 (41.7)
Diuretics, *n* (%)	2,466 (55.7)	2,635 (75.7)

*DM, diabetes mellitus; CKD, chronic kidney diseases; AF, atrial fibrillation; COPD, chronic obstructive pulmonary disease; PCI, percutaneous coronary intervention; DES, drug eluting stents; BES, bare metal stent; NT-proBNP, N terminal pro B type natriuretic peptide; eGFR, estimated glomerular filtration rate; HGB, hemoglobin; ACEI/ARB, angiotensin-converting enzyme inhibitor/angiotensin receptor blocker; CCB, calcium channel blockers*.

More details of patient characteristics at baseline according to the cutoff of the Cl^−^ level in serum (98 mmol/L) are shown in Table 2. A total of 8,243 CAD patients with CHF upon hospital admission were divided into two groups. Hypochloremia accounted for 10.2% (*n* = 484) of cases in CIN-I and 20.1% (*n* = 698) of cases in MIMIC-III (Chi-square test: *P* <0.001). Among patients with hypochloremia in these two databases, patients with hypochloremia were negatively associated with an increased serum level of sodium ions (Na^+^) and positively associated with the prevalence of DM, CKD, and AMI. The prevalence of diuretic use was higher among patients with hypochloremia than in those not suffering from hypochloremia. Nevertheless, there was no significant difference between the prevalence of atrial fibrillation or stroke and the serum level of Cl^−^.

**Table 2:** Baseline characteristics of the patients with coronary artery disease (CAD) and congestive heart failure (CHF).

**Characteristic**	**CIN-I**			**MIMIC-III**		
	**Non-hypochloremia**	**Hypochloremia**	***P*-value**	**Non-hypochloremia**	**Hypochloremia**	***P*-value**
	**(*N* = 4,278)**	**(*N* = 484)**		**(*N* = 2,783)**	**(*N* = 698)**	
**Demographic characteristics**						
Female, *n* (%)	1,023 (23.9)	150 (31.0)	0.001	1,051 (37.8)	293 (42.0)	0.045
Age, years, mean (SD)	65.3 (10.9)	67.1 (11.1)	0.001	74.5 (11.7)	73.6 (11.3)	0.064
**Medical history**						
Anemia, *n* (%)	1,957 (46.3)	235 (49.0)	0.281	1,972 (70.9)	514 (73.7)	0.148
AMI, *n* (%)	2,044 (47.8)	259 (53.5)	0.019	1,452 (52.2)	337 (48.3)	0.072
DM, *n* (%)	1,443 (33.7)	216 (44.6)	<0.001	1,102 (39.6)	369 (52.9)	<0.001
CKD, *n* (%)	1,801 (42.4)	262 (54.6)	<0.001	687 (24.7)	259 (37.1)	<0.001
Hypertension, *n* (%)	2,510 (58.7)	281 (58.1)	0.833	1,155 (41.5)	242 (34.7)	0.001
AF, *n* (%)	318 (7.4)	42 (8.7)	0.373	1,205 (43.3)	330 (47.3)	0.064
COPD, *n* (%)	65 (1.5)	14 (2.9)	0.04	149 (5.4)	50 (7.2)	0.08
Stroke, *n* (%)	361 (8.4)	33 (6.8)	0.255	312 (11.2)	62 (8.9)	0.088
**Procedure**, ***n*** **(%)**						
PCI	3,097 (72.4)	361 (74.6)	0.331	549 (21.6)	105 (16.6)	0.006
DES	2,944 (68.8)	349 (72.1)	0.152	266 (10.5)	54 (8.5)	0.172
BES	80 (1.9)	8 (1.7)	0.874	310 (12.2)	59 (9.3)	0.052
**Laboratory tests**						
NT-proBNP, pg/ml, mean (SD)	4,747.0 (9,451.1)	7,410.6 (9,526.5)	<0.001	13,216.4 (16,164.6)	15,350.9 (17,932.9)	0.168
HGB, g/L, mean (SD)	127.3 (20.3)	127.2 (22.3)	0.959	115.7 (21.3)	114.0 (20.8)	0.059
eGFR, mL/min/1.73 m^2^ [mean (SD)]	65.9 (27.0)	59.5 (29.8)	<0.001	57.6 (31.2)	46.4 (32.9)	<0.001
K, mmol/L, mean (SD)	3.84 (0.52)	3.87 (0.65)	0.211	4.37 (0.79)	4.55 (0.93)	<0.001
Na, mmol/L, mean (SD)	138.7 (3.1)	133.5 (4.5)	<0.001	139.2 (3.7)	133.1 (4.8)	<0.001
Ca, mmol/L, mean (SD)	2.20 (0.14)	2.20 (0.17)	0.485	2.15(0.21)	2.19(0.19)	<0.001
**Medications**						
ACEI/ARB, *n* (%)	1,902 (47.7)	214 (49.1)	0.606	1,588 (57.1)	361 (51.7)	0.012
Beta-blockers, *n* (%)	3,173 (79.5)	338 (77.5)	0.364	2,325 (83.5)	578 (82.8)	0.682
CCB, *n* (%)	715 (17.9)	70 (16.1)	0.368	666 (23.9)	170 (24.4)	0.853
Statin, *n* (%)	3,636 (91.1)	388 (89.0)	0.171	2,112 (75.9)	508 (72.8)	0.098
Aspirin, *n* (%)	3,495 (87.6)	366 (83.9)	0.038	2,252 (80.9)	575 (82.4)	0.408
Clopidogrel, *n* (%)	3,260 (81.7)	368 (84.4)	0.181	1,156 (41.5)	296 (42.4)	0.709
Diuretics, *n* (%)	2,179 (54.6)	287 (65.8)	<0.001	2,147 (77.1)	488 (69.9)	<0.001

*DM, diabetes mellitus; CKD, chronic kidney diseases; AF, atrial fibrillation; COPD, chronic obstructive pulmonary disease; PCI, percutaneous coronary intervention; DES, drug eluting stents; BES, bare metal stent; NT-proBNP, N terminal pro B type natriuretic peptide; eGFR, estimated glomerular filtration rate; HGB, hemoglobin; ACEI/ARB, angiotensin-converting enzyme inhibitor/angiotensin receptor blocker; CCB, calcium channel blockers*.

### Cl^–^ Levels and Clinical Endpoints

**Figure 3:**
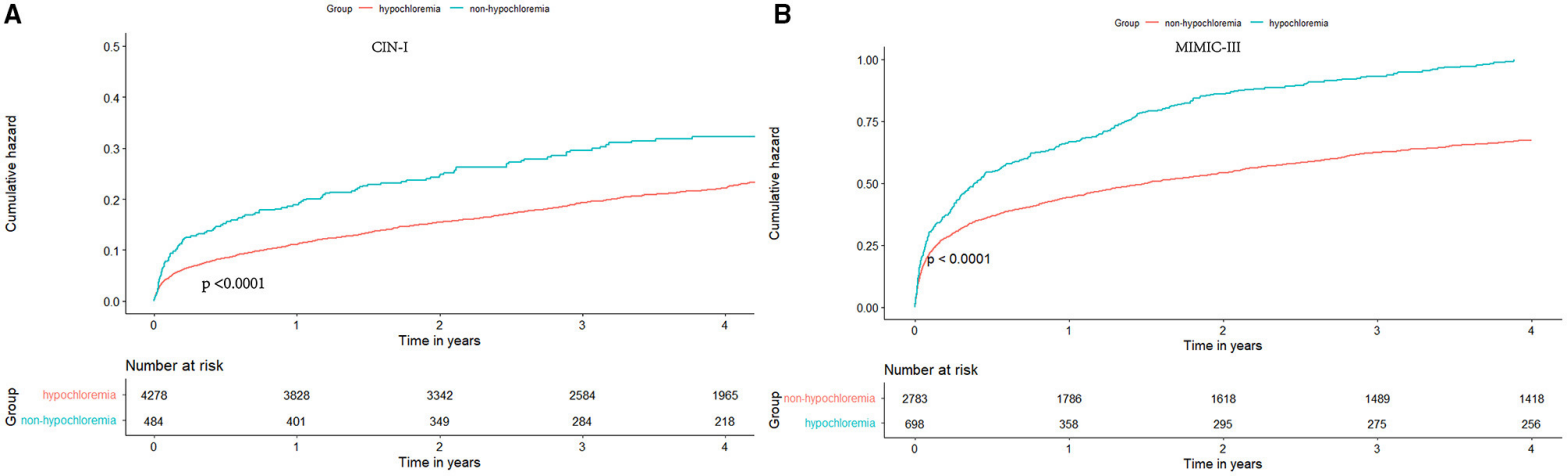
Kaplan-Meier curves for long-term all-cause mortality (ACM) of hypochloremia. **(A)** CIN-I; **(B)** MIMIC-III.

**Figure 4:**
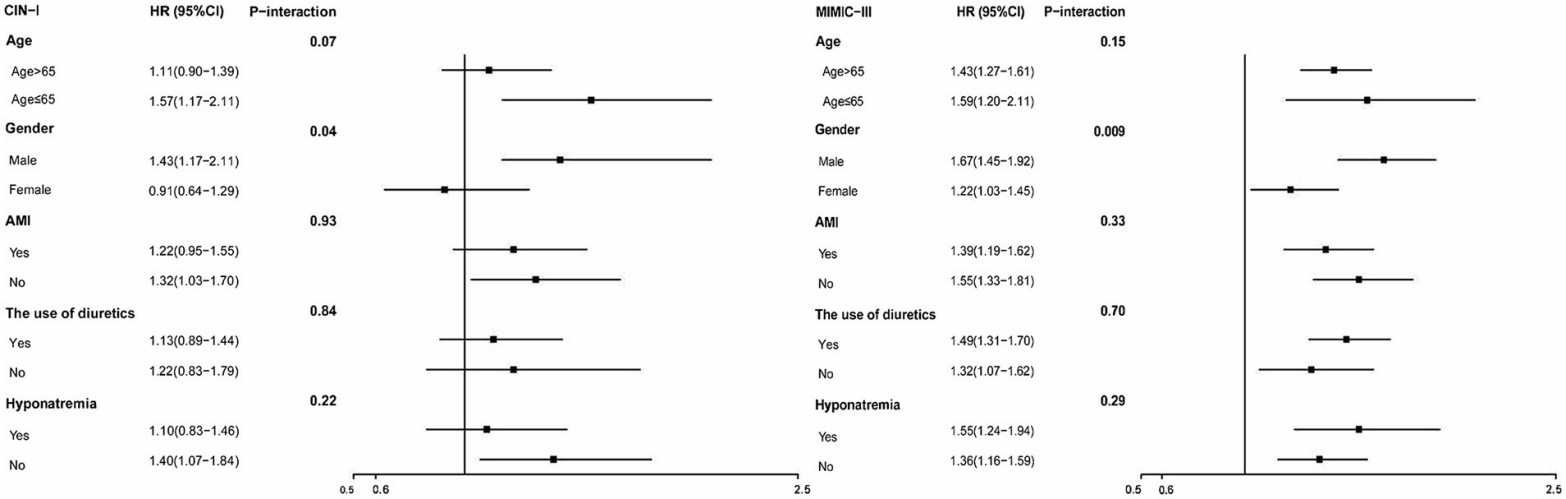
Hazard ratios for long-term ACM in different subgroups (hypochloremia vs. non-hypochloremia).

During a median follow-up of 3.7 (range, 2.1–5.9) years, 1,123 (23.6%) patients died in CIN-I. During a median follow-up of 3.2 (range, 0.2–4) years, 1,807 (51.9%) patients died in MIMIC-III. Moreover, 330 patients (6.9%) died in CIN-I and 330 patients (27.3%) died in MIMIC-III from all causes within 90-day follow-up after hospital admission. When the serum level of Cl^−^ was low, there was an inverse association between the hazard ratio (*HR*) for the endpoint and serum Cl^−^ level (Figure 3; *P* for non-linear < 0.001). Patients suffering from hypochloremia carried a higher mortality than those not suffering from hypochloremia (90-day ACM: 11.8 vs. 6.4%, *P* < 0.001 in CIN-I; 33.2 vs. 25.8%, *P* < 0.001 in MIMIC-III; long-term ACM: 30.4 vs. 22.8%, *P* < 0.001 in CIN-I; 63.3 vs. 49.0%, *P* < 0.001 in MIMIC-III). Kaplan–Meier analyses illustrated that hypochloremia may be related to worsening of 90-day ACM and long-term ACM in the two databases (*P* < 0.0001; log-rank test) (Figure 4). Cox proportional hazards models were used to evaluate the relationships between ACM and hypochloremia. These models indicated that hypochloremia was associated with a higher risk of 90-day ACM even after full adjustment of major confounders (*HR*, 1.69, 95% *CI* 1.27–2.25, *P* < 0.001 in CIN-I; *HR*, 1.36, 95% *CI* 1.17–1.59, *P* < 0.001 in MIMIC-III) as compared with a normal serum level of Cl^−^ (Table 3A). Importantly, these data suggested that the serum Cl^−^ level upon hospital admission was independently related to long-term ACM. In the CIN-I cohort, hypochloremia was associated with a higher risk of long-term ACM compared with a normal serum level of Cl^−^ (model 1: HR 1.37, 95% *CI* 1.15–1.63, *P* < 0.001; model 2: *HR* 1.29, 95% *CI* 1.09–1.54, *P* = 0.004; model 3: *HR* 1.26, 95% *CI* 1.06–1.50, *P* = 0.009). In the MIMIC-III cohort, hypochloremia was at virtually the approximate risk of mortality (model 1: *HR* 1.48, 95% *CI* 1.33–1.64, *P* < 0.001; model 2: *HR* 1.52, 95% *CI* 1.36–1.69, *P* < 0.001; model 3: *HR* 1.48, 95% *CI* 1.32–1.66, *P* < 0.001) (Table 3B).

**Table 3:** The association between hypochloremia and mortality in different models.

**A. Cox proportional hazard ratios (** ***HR*** **s) for 90-day all-cause mortality (ACM) in different models**						
**Database**	**Group**	***N***	**Events**, ***n*** **(%)**	**90-day ACM HR, 95%Cl**, ***p*****-value**		
				Model 1^*^	**Model 2^$^**	**Model 3 ^§^**
**CIN-I**						
	Non-hypochloremia	4,278	273 (6.4%)	ref	ref	ref
	Hypochloremia	484	57 (11.8%)	1.89 (1.42–2.51), <0.001	1.80 (1.35–2.40), <0.001	1.69 (1.27–2.25), <0.001
**MIMIC-III**						
	Non-hypochloremia	2,783	717(25.8%)	ref	ref	ref
	Hypochloremia	698	232(33.2%)	1.35 (1.16–1.56), <0.001	1.40 (1.20–1.62), <0.001	1.36 (1.17–1.59), <0.001
**B. Cox proportional** ***HR*****s for long-term ACM in different models**						
**Database**	**Group**	***N***	**Events**, ***n*** **(%)**	**Long-term ACM HR, 95%Cl**, ***p*****-value**		
				Model 1^*^	**Model 2^$^**	**Model 3^§^**
**CIN-I**						
	Non-hypochloremia	4,278	976 (22.8%)	ref	ref	ref
	Hypochloremia	484	147 (30.4%)	1.37 (1.15–1.63), <0.001	1.29 (1.09–1.54), 0.004	1.26 (1.06–1.50), 0.009
**MIMIC-III**						
	Non-hypochloremia	2,783	1,365 (49.0%)	ref	ref	ref
	Hypochloremia	698	442 (63.3%)	1.48 (1.33–1.64), <0.001	1.52 (1.36–1.69), <0.001	1.48 (1.32–1.66), <0.001

* *Unadjusted*.

$ *Adjusted for age, gender*.

§ *Adjusted for full multivariate: age, gender, hypotension, anemia, DM, AF, CKD, COPD, stroke, and acute myocardial infarction (AMI)*.

**Figure 5:**
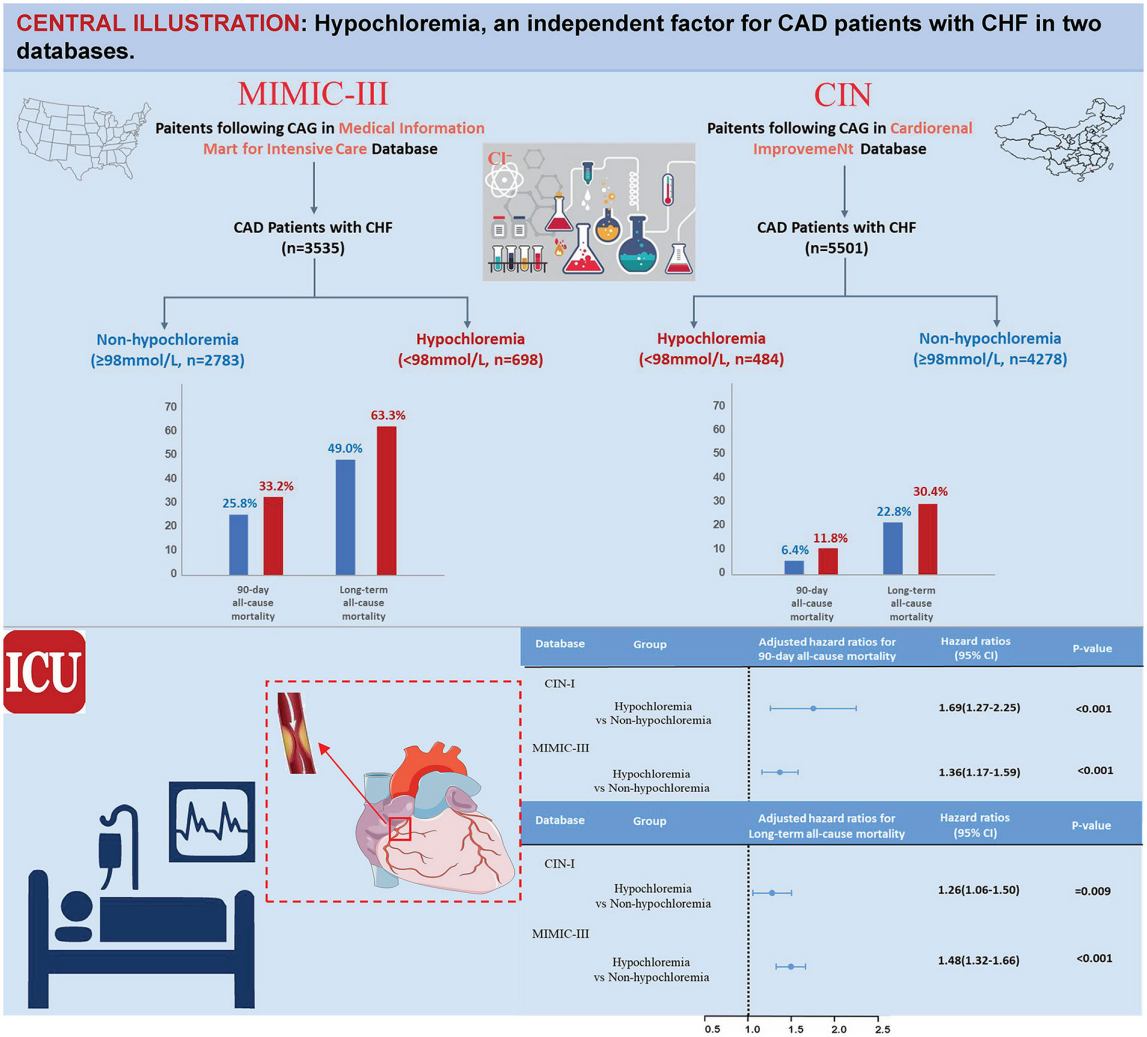
Central illustration.

For subgroups, Cox regression analysis demonstrated that hypochloremia was associated with a consistent risk of mortality across dichotomized subgroups (age, AMI, and diuretic use). However, the prespecified subgroup analyses revealed the association of hypochloremia with long-term ACM to be attenuated slightly in the women of the two databases (*P* interaction < 0.001) (Figure 5).

## Discussion

Our study is the first systematic exploration of the prevalence and mortality of hypochloremia among CAD patients with CHF in two databases. Our study indicated that ~10% of CAD patients with CHF were complicated with hypochloremia in the CIN-I database and ~20% in the relatively high-risk MIMIC-III database. Among those patients, hypochloremia was independently associated with higher short- and long-term ACM. The impact of hypochloremia was consistent and independent of the presence of AMI or use of diuretics. In addition, the association of hypochloremia with long-term ACM was attenuated slightly in the women of the two databases.

Epidemiological data have demonstrated that HF is a global disease (20). The most common reason for HF is CAD (21). Physicians need to use indices to evaluate the outcome among CAD patients with CHF. Chloride is the most plentiful anion in the extracellular fluid (12). Some studies have suggested that regulation of renin secretion from the macula densa in the kidneys may be reliant on Cl^−^ (22). The latter is crucial in acid–base balance, muscle activity, and immune regulation (23). Non-potassium-sparing diuretics are used widely (particularly among patients with HF). These agents primarily inhibit the Na^+^-potassium ion–Cl^−^ cotransporter, which leads inevitably to the excessive wastage of Cl^−^. The prognostic value of Cl^−^ on HF was reported by Grodin and colleagues, who stated that hypochloremia upon hospital admission was associated with poor long-term survival (8). Li et al. studied the relationship of Cl^−^ and short-term mortality in all CCU patients who have complex conditions and many confounding factors (6). However, evidence of the prognostic value of Cl^−^ for CAD patients with CHF is lacking. We revealed that the Cl^−^ concentration is an independent factor of short- and long-term mortality for CAD patients with CHF in two databases.

The exact mechanisms underlying the relationship between hypochloremia and CAD with CHF are not known, but three hypotheses have been proposed. First, the reason why hypochloremia is associated with short- and long-term mortality is that a low concentration of Cl^−^ might upregulate the expression of proinflammatory cytokines. This action may accelerate inflammation (24), which is a well-known predictor of poor outcomes and mortality for CAD (25–27) and HF (28). Second, Cl^−^ has a critical role in regulatory pathways central to physiological stability. For example, a low concentration of Cl^−^ means a high anion gap and high renin level (29–31), which is related to higher blood pressure (32) and stimulates HF directly (33). Additionally, several Cl^−^ channels with important regulatory functions have been identified or characterized further recently (23, 34). Third, according to our results, hypochloremia could be considered a marker of clinical complexity because hypochloremia is observed more often in older patients with DM, CKD, or AMI, which eventually leads to a significantly increased mortality risk (35–39).

In addition, a study had reported that hypochloremia results in hyponatremia and that they are significantly associated with each other and increased mortality risk (9). The increase in the prevalence of hyponatremia reported in that study was consistent with our findings, but we did not find that hyponatremia affected death. This may have been because our study population had CHD with CHF. Furthermore, long-term ACM was attenuated slightly in the women of the two databases. However, it seems that this phenomenon is not reported previously. More randomized controlled trials and basic research are needed to validate these phenomena.

Two databases were selected for our study. There were certain differences between the two databases. A higher prevalence of hypochloremia and mortality was observed in the relatively high-risk MIMIC-III database, which was in line with clinical concepts. The same connection exists even in relatively high-risk groups, which indicates that our conclusion was applicable to a wider range of people. Interestingly, in the MIMIC database, hypochloremia seemed to have a more substantial effect on long-term ACM than short-term ACM.

Our study had three main limitations. First, our population was from two single-center retrospective cohort studies with a selection bias. However, the MIMIC-III database has been used widely and shown to be of high quality. In addition, we added a large sample database from China. Therefore, our study included sufficient samples to make it representative. Second, information on cause-specific death was not presented here, and it was difficult to examine a significant correlation between hypochloremia and cause-specific death. ACM is an important endpoint that clinicians concentrate upon commonly. Third, Cl^−^ levels were extracted only upon hospital admission without assessment of their changes after hospital discharge.

## Conclusions

Our results suggested that hypochloremia could be a risk-assessment tool for primary-care clinicians. The serum Cl^−^ level may represent a therapeutic target rather than being a marker of disease severity. Early adequate assessment of hypochloremia and active supplementation with Cl^−^ (such as oral and intravenous supplements of concentrated sodium chloride) may help to improve the prognosis of patients with CAD and CHF. Hence, evaluation of the mechanism of the association between hypochloremia and mortality in CAD patients with CHF, and whether the mortality risk decreases after treating hypochloremia, merit investigation. Also, more emphasis should be laid on female patients suffering from CAD with CHF. Large-scale multi-center prospective studies are needed to verify our conclusions.

## Data Availability Statement

The original contributions presented in the study are included in the article/Supplementary Materials, further inquiries can be directed to the corresponding authors.

## Ethics Statement

Ethical review and approval was not required for the study on human participants in accordance with the local legislation and institutional requirements. Written informed consent for participation was not required for this study in accordance with the national legislation and the institutional requirements.

## Author Contributions

YLiu, NT, DH, and JC designed the study, reviewed, interpreted, and checked clinical data. YLia, CC, JL, and XH collected and reviewed clinical and laboratory data. SC, HH, LQ, QL, and WL analyzed data. SC, HH, YLiu, LQ, and BW performed the statistical analysis. JL, HH, YLiu, KB, QL, and JLL drafting or revising of the manuscript. All authors contributed to the article and approved the submitted version.

## Funding

This research was funded and supported by the Beijing Lisheng Cardiovascular Health Foundation Pilot Fund (No. LHJJ20141751); The National Science Foundation of China (No. 81970311 and No. 82070360); Study on the function and mechanism of the potential target for early warning of cardiorenal syndrome after acute myocardial infarction based on transformism (DFJH201919); Clinical Medicine Research Fund of Guangdong Province (2019ZX01)

## Conflict of Interest

The authors declare that the research was conducted in the absence of any commercial or financial relationships that could be construed as a potential conflict of interest.

## Publisher's Note

All claims expressed in this article are solely those of the authors and do not necessarily represent those of their affiliated organizations, or those of the publisher, the editors and the reviewers. Any product that may be evaluated in this article, or claim that may be made by its manufacturer, is not guaranteed or endorsed by the publisher.
