# Supplementary material for: Prevalence and Mortality of Hypochloremia Among Patients Suffering From Coronary Artery Disease and Congestive Heart Failure: An Analysis of Patients in CIN-I and MIMIC-III Databases
Source: Front Med (Lausanne). 2021 Dec 21;8:769646. doi: 10.3389/fmed.2021.769646 (PMC8724045; doi:10.3389/fmed.2021.769646)
Supplement: Supplementary file 1 [file Table_1.DOCX]

**Supplement Table 1 Logistic model for 90-day mortality.**

| Database | Group | 90-day ACM | | |
| --- | --- | --- | --- | --- |
|  |  | OR, 95%Cl, p-value | | |
| CIN-I |  | Model 1* | Model 2^$^ | Model 3^§^ |
|  | Non-hypochloremia | ref | ref | ref |
|  | Hypochloremia | 1.96 (1.44-2.63), <0.001 | 1.88 (1.38-2.53), <0.001 | 1.77(1.28-2.40), <0.001 |
| MIMIC-III | |  |  |  |
|  | Non-hypochloremia | ref | ref | ref |
|  | Hypochloremia | 1.43 (1.20-1.71), <0.001 | 1.53 (1.27-1.84), <0.001 | 1.49(1.22-1.81), <0.001 |

* Unadjusted

^$^ Adjusted for age, gender;

^§^Adjusted for full multivariate: age, gender, hypotension, anemia, diabetes mellitus, atrial fibrillation, chronic kidney diseases, chronic obstructive pulmonary disease, stroke and acute myocardial infarction.
